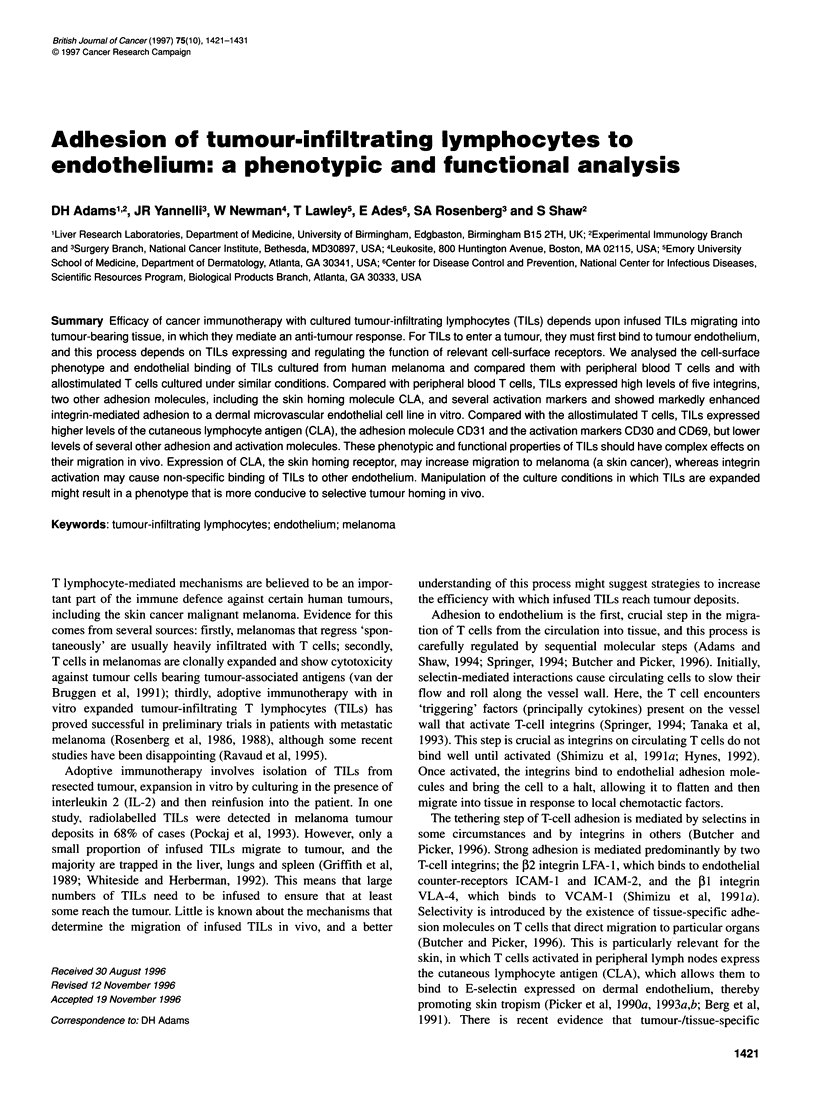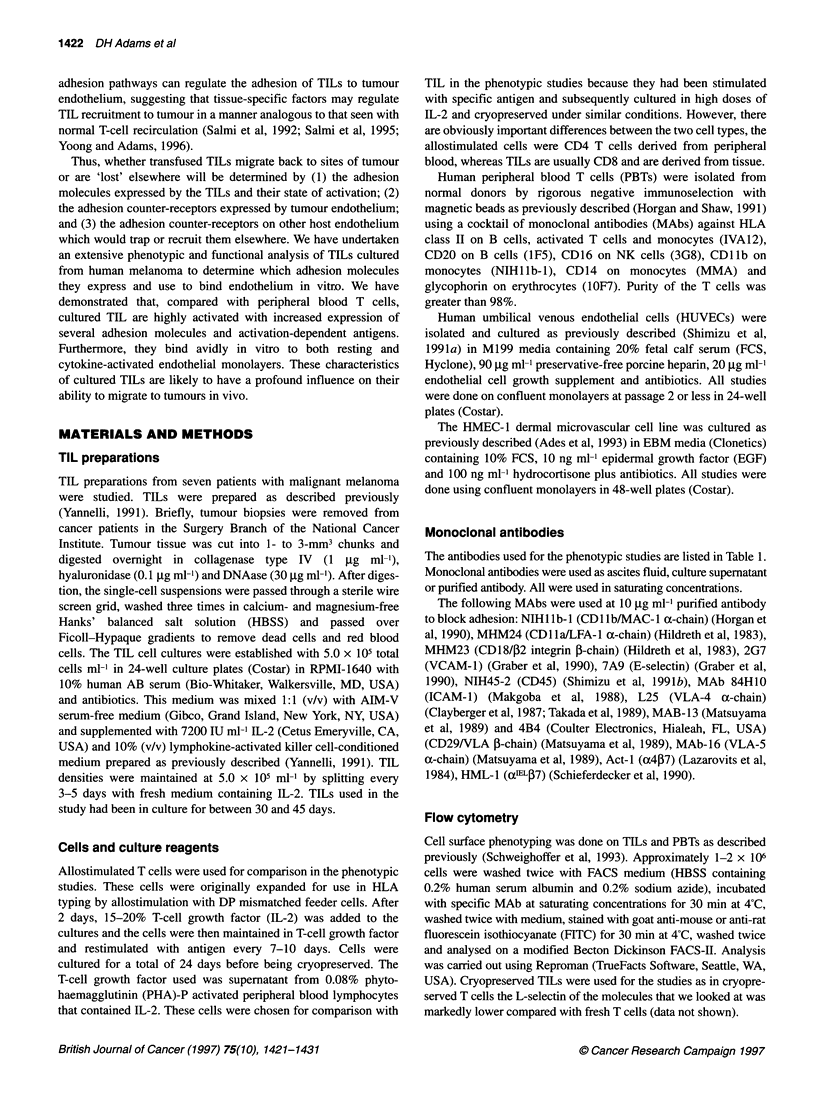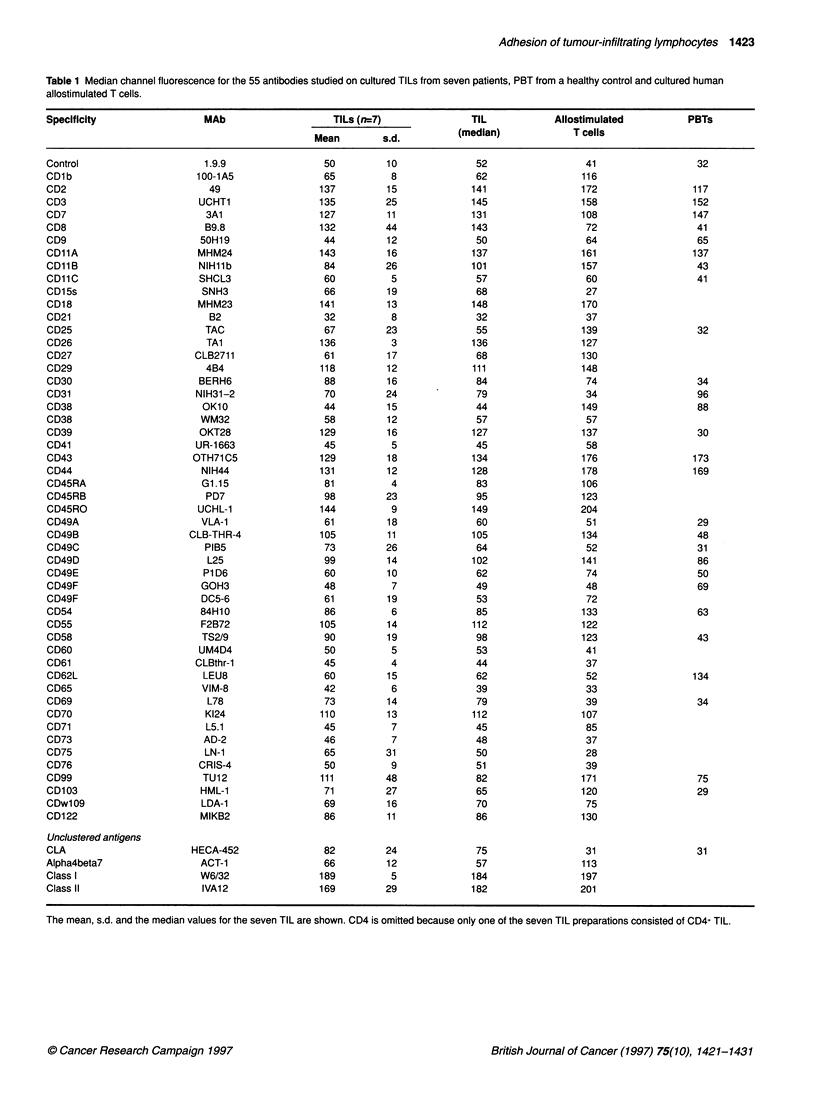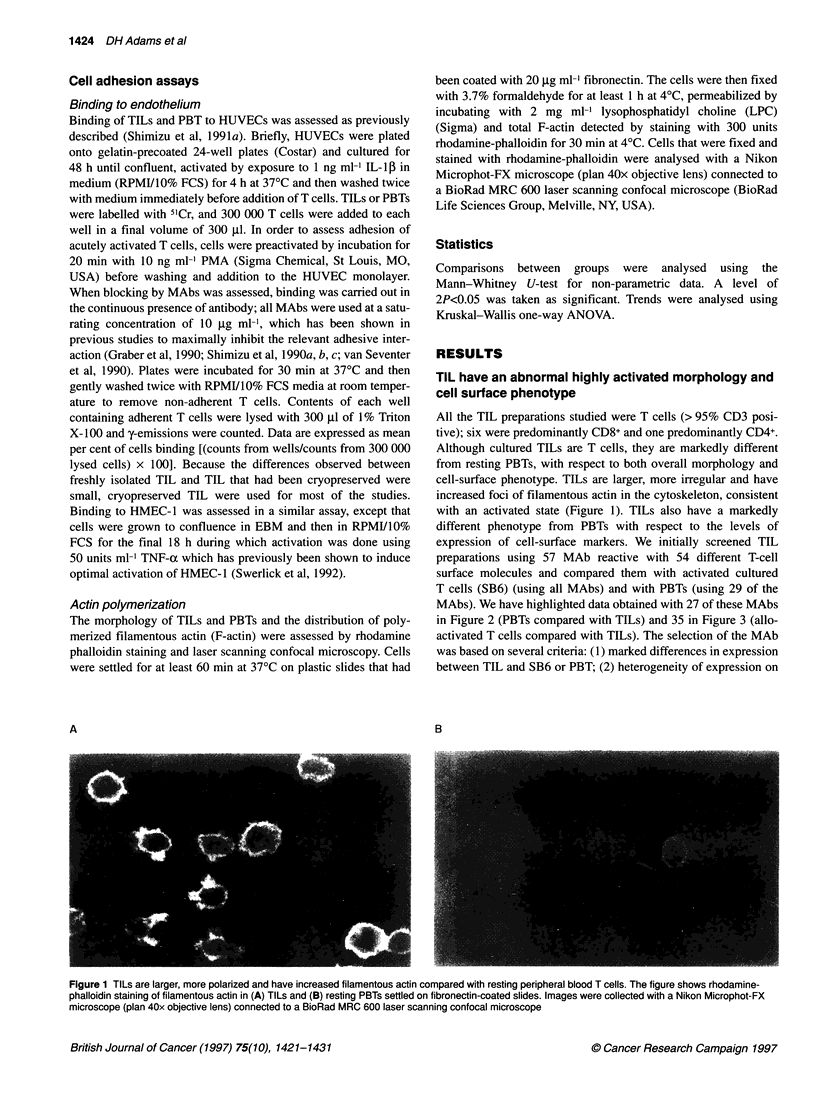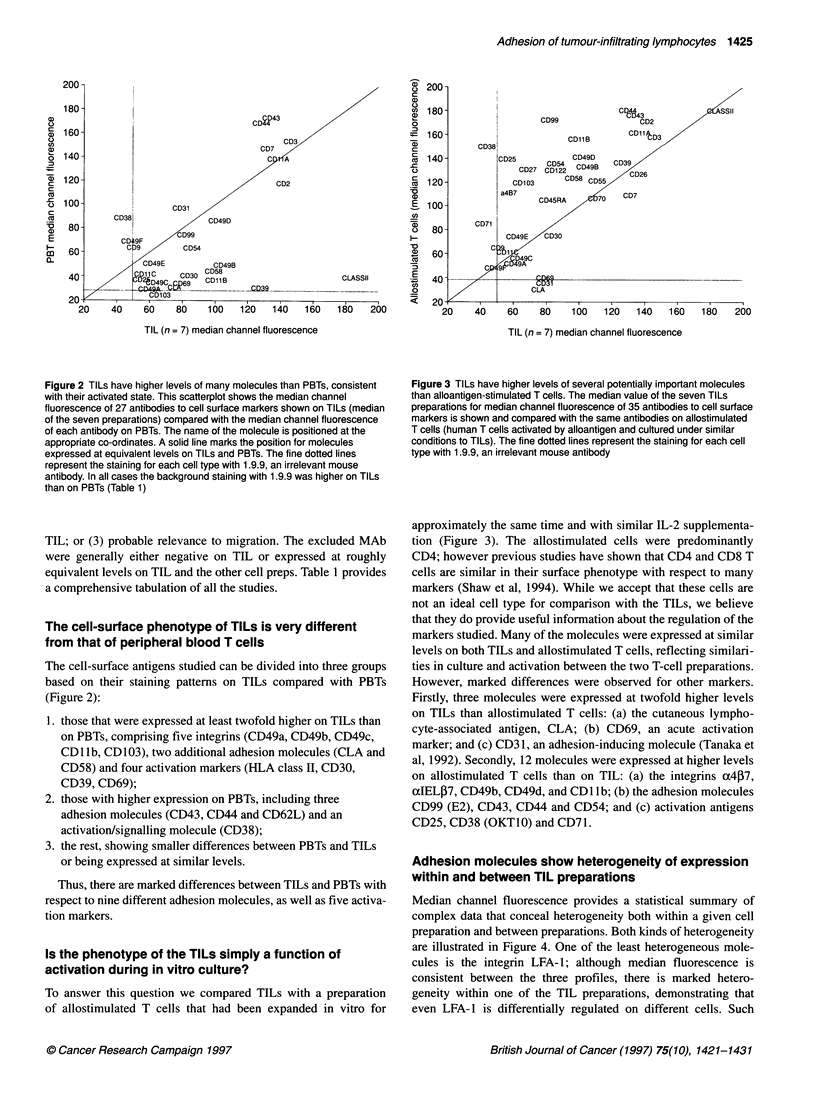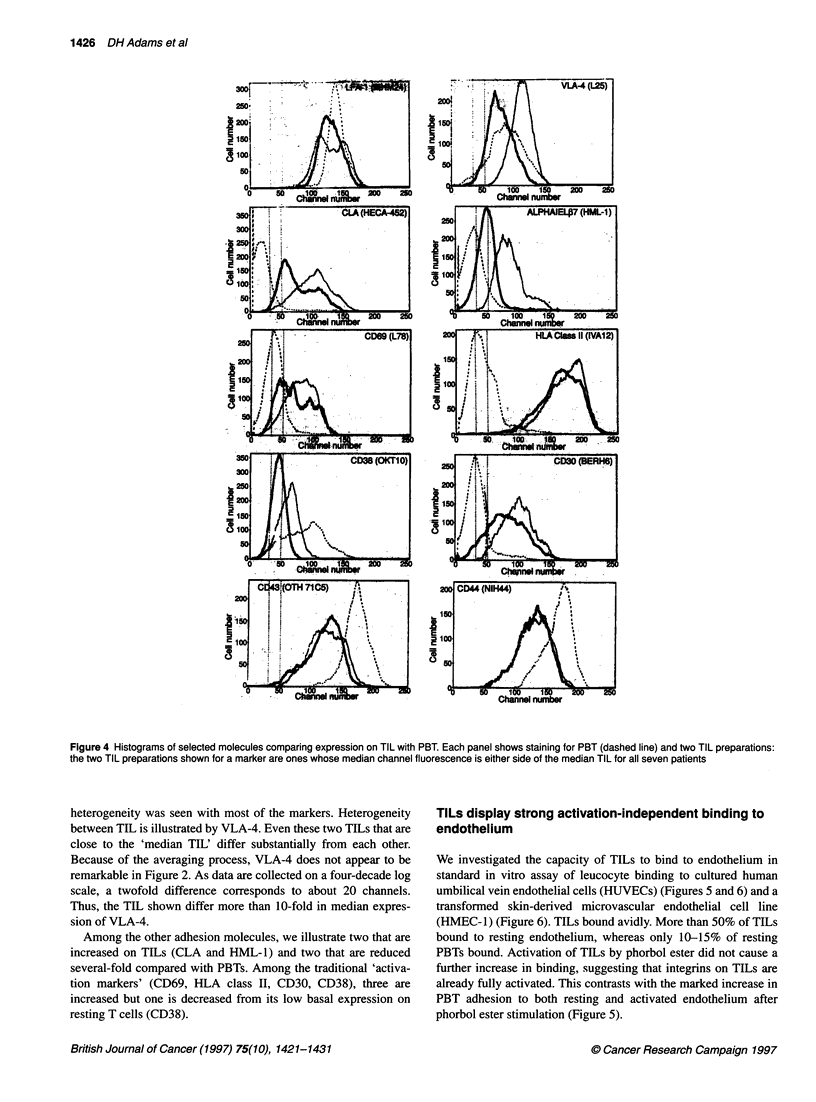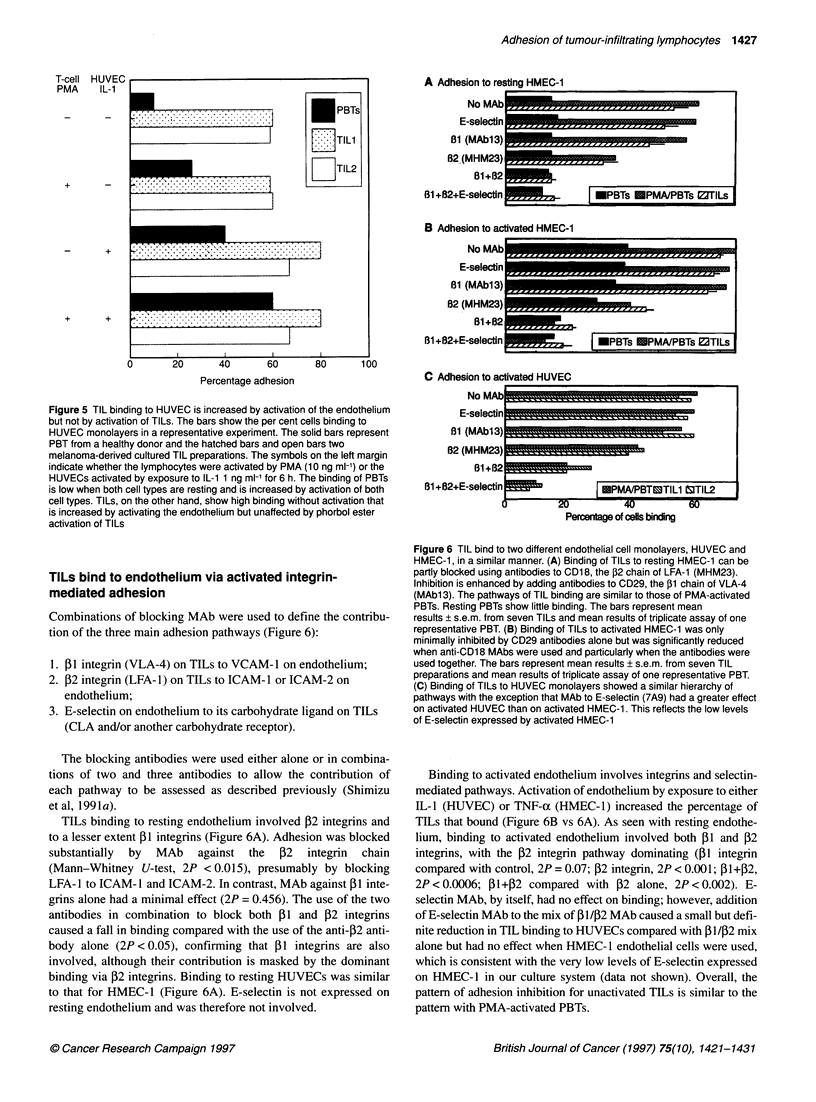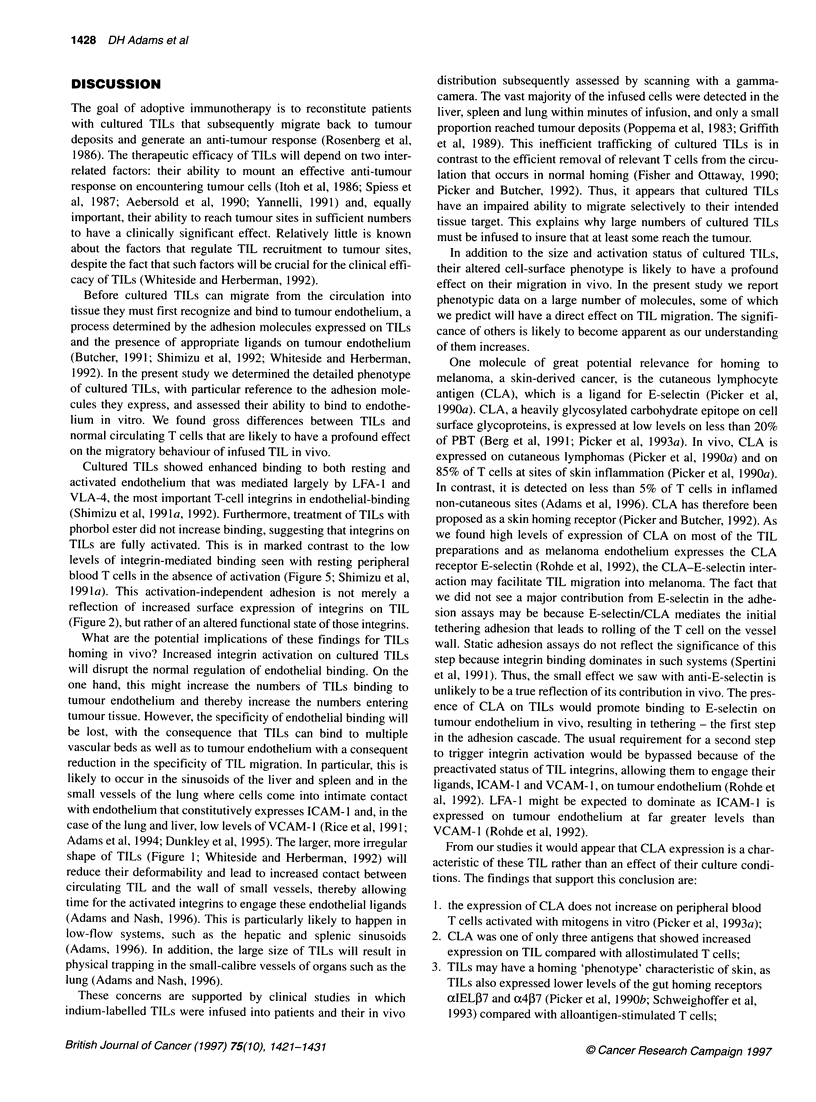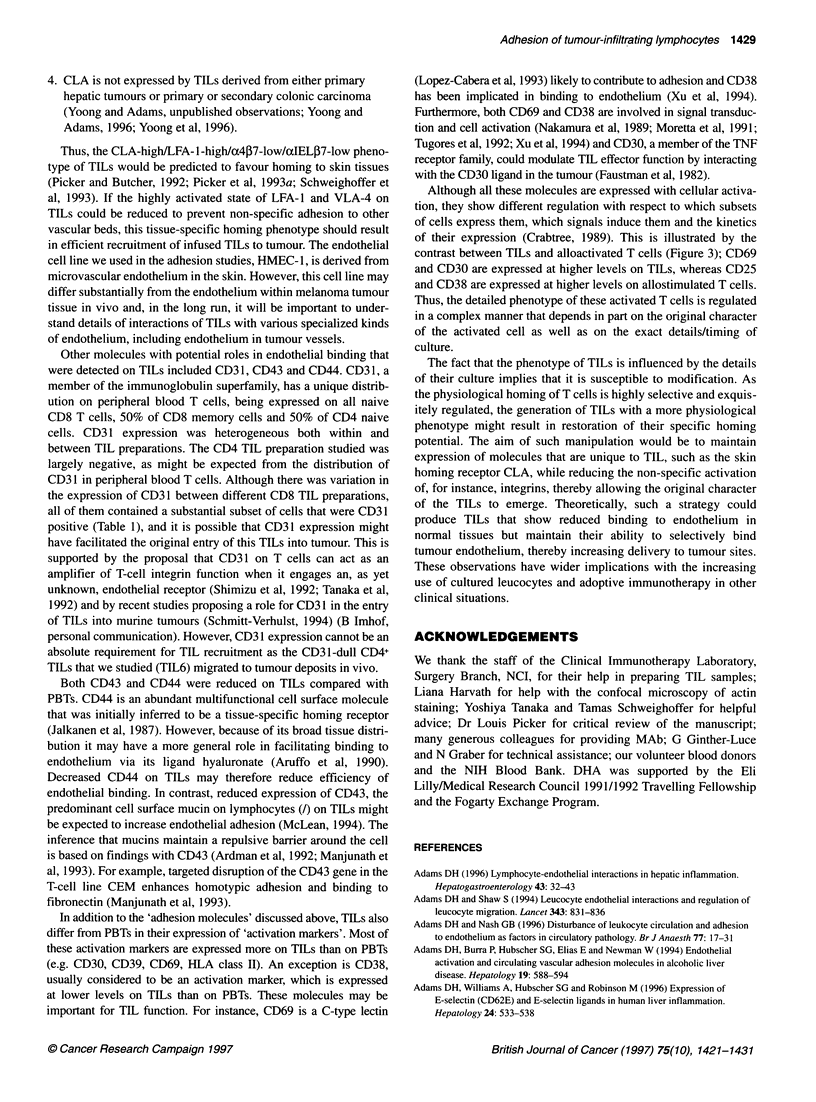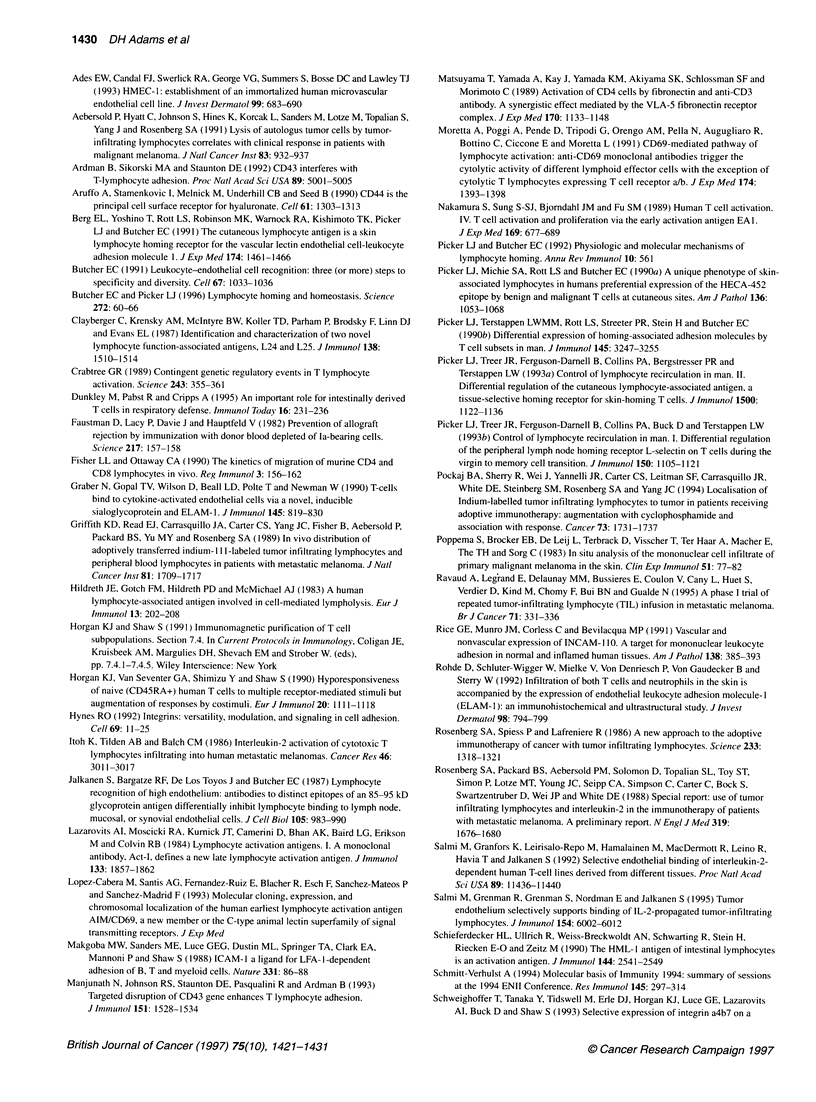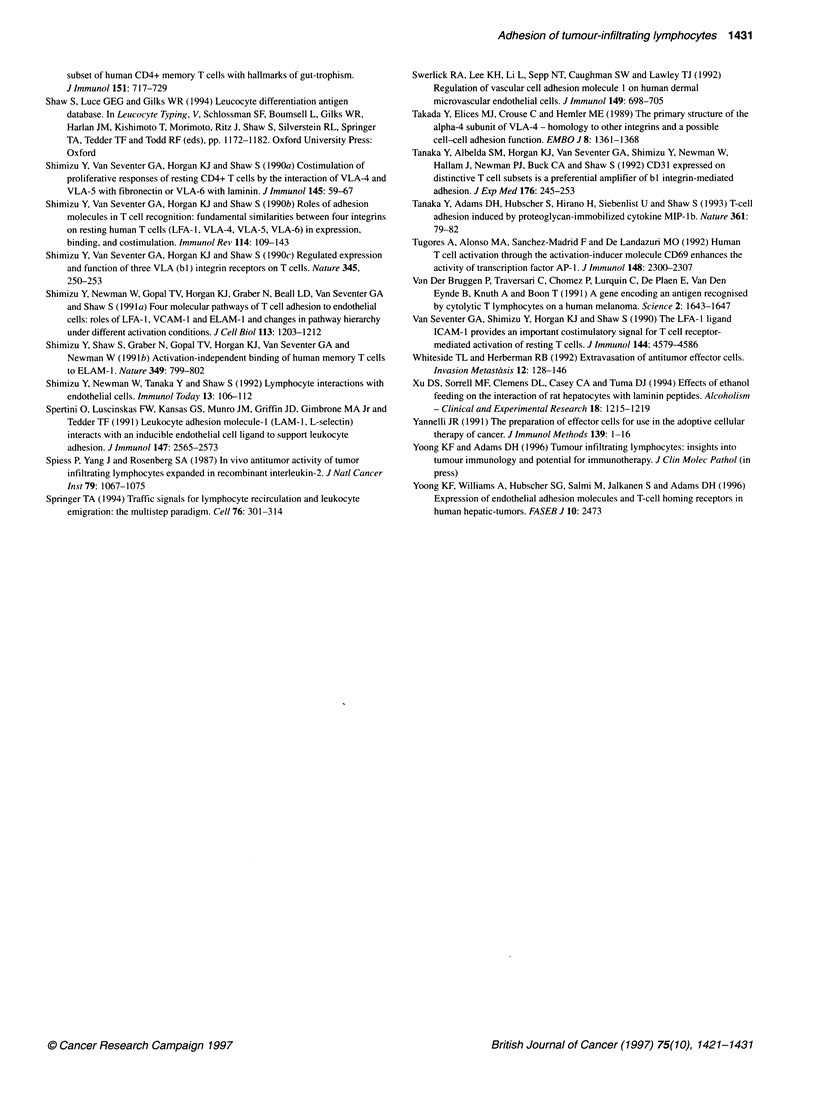# Adhesion of tumour-infiltrating lymphocytes to endothelium: a phenotypic and functional analysis.

**DOI:** 10.1038/bjc.1997.245

**Published:** 1997

**Authors:** D. H. Adams, J. R. Yannelli, W. Newman, T. Lawley, E. Ades, S. A. Rosenberg, S. Shaw

**Affiliations:** Department of Medicine, University of Birmingham, Edgbaston, UK.

## Abstract

**Images:**


*British Journal of Cancer* (1997) **75**(10), 1421–1431
© 1997 Cancer Research Campaign

### Adhesion of tumour-infiltrating lymphocytes to endothelium: a phenotypic and functional analysis

**DH Adams[1,2], JR Yannelli[3], W Newman[4], T Lawley[5], E Ades[6], SA Rosenberg[3] and S Shaw[2]**

[1]Liver Research Laboratories, Department of Medicine, University of Birmingham, Edgbaston, Birmingham B15 2TH, UK; [2]Experimental Immunology Branch and [3]Surgery Branch, National Cancer Institute, Bethesda, MD30897, USA; [4]Leukosite, 800 Huntington Avenue, Boston, MA 02115, USA; [5]Emory University School of Medicine, Department of Dermatology, Atlanta, GA 30341, USA; [6]Center for Disease Control and Prevention, National Center for Infectious Diseases, Scientific Resources Program, Biological Products Branch, Atlanta, GA 30333, USA

**Summary** Efficacy of cancer immunotherapy with cultured tumour-infiltrating lymphocytes (TILs) depends upon infused TILs migrating into tumour-bearing tissue, in which they mediate an anti-tumour response. For TILs to enter a tumour, they must first bind to tumour endothelium, and this process depends on TILs expressing and regulating the function of relevant cell-surface receptors. We analysed the cell-surface phenotype and endothelial binding of TILs cultured from human melanoma and compared them with peripheral blood T cells and with allostimulated T cells cultured under similar conditions. Compared with peripheral blood T cells, TILs expressed high levels of five integrins, two other adhesion molecules, including the skin homing molecule CLA, and several activation markers and showed markedly enhanced integrin-mediated adhesion to a dermal microvascular endothelial cell line in vitro. Compared with the allostimulated T cells, TILs expressed higher levels of the cutaneous lymphocyte antigen (CLA), the adhesion molecule CD31 and the activation markers CD30 and CD69, but lower levels of several other adhesion and activation molecules. These phenotypic and functional properties of TILs should have complex effects on their migration in vivo. Expression of CLA, the skin homing receptor, may increase migration to melanoma (a skin cancer), whereas integrin activation may cause non-specific binding of TILs to other endothelium. Manipulation of the culture conditions in which TILs are expanded might result in a phenotype that is more conducive to selective tumour homing in vivo.

**Keywords**: tumour-infiltrating lymphocytes; endothelium; melanoma

T lymphocyte-mediated mechanisms are believed to be an important part of the immune defence against certain human tumours, including the skin cancer malignant melanoma. Evidence for this comes from several sources: firstly, melanomas that regress 'spontaneously' are usually heavily infiltrated with T cells; secondly, T cells in melanomas are clonally expanded and show cytotoxicity against tumour cells bearing tumour-associated antigens (van der Bruggen et al, 1991); thirdly, adoptive immunotherapy with in vitro expanded tumour-infiltrating T lymphocytes (TILs) has proved successful in preliminary trials in patients with metastatic melanoma (Rosenberg et al, 1986, 1988), although some recent studies have been disappointing (Ravaud et al, 1995).

Adoptive immunotherapy involves isolation of TILs from resected tumour, expansion in vitro by culturing in the presence of interleukin 2 (IL-2) and then reinfusion into the patient. In one study, radiolabelled TILs were detected in melanoma tumour deposits in 68% of cases (Pockaj et al, 1993). However, only a small proportion of infused TILs migrate to tumour, and the majority are trapped in the liver, lungs and spleen (Griffith et al, 1989; Whiteside and Herberman, 1992). This means that large numbers of TILs need to be infused to ensure that at least some reach the tumour. Little is known about the mechanisms that determine the migration of infused TILs in vivo, and a better understanding of this process might suggest strategies to increase the efficiency with which infused TILs reach tumour deposits.

*Received 30 August 1996*
*Revised 12 November 1996*
*Accepted 19 November 1996*

*Correspondence to:* DH Adams

Adhesion to endothelium is the first, crucial step in the migration of T cells from the circulation into tissue, and this process is carefully regulated by sequential molecular steps (Adams and Shaw, 1994; Springer, 1994; Butcher and Picker, 1996). Initially, selectin-mediated interactions cause circulating cells to slow their flow and roll along the vessel wall. Here, the T cell encounters 'triggering' factors (principally cytokines) present on the vessel wall that activate T-cell integrins (Springer, 1994; Tanaka et al, 1993). This step is crucial as integrins on circulating T cells do not bind well until activated (Shimizu et al, 1991*a*; Hynes, 1992). Once activated, the integrins bind to endothelial adhesion molecules and bring the cell to a halt, allowing it to flatten and then migrate into tissue in response to local chemotactic factors.

The tethering step of T-cell adhesion is mediated by selectins in some circumstances and by integrins in others (Butcher and Picker, 1996). Strong adhesion is mediated predominantly by two T-cell integrins; the β2 integrin LFA-1, which binds to endothelial counter-receptors ICAM-1 and ICAM-2, and the β1 integrin VLA-4, which binds to VCAM-1 (Shimizu et al, 1991*a*). Selectivity is introduced by the existence of tissue-specific adhesion molecules on T cells that direct migration to particular organs (Butcher and Picker, 1996). This is particularly relevant for the skin, in which T cells activated in peripheral lymph nodes express the cutaneous lymphocyte antigen (CLA), which allows them to bind to E-selectin expressed on dermal endothelium, thereby promoting skin tropism (Picker et al, 1990*a*, 1993*a*,*b*; Berg et al, 1991). There is recent evidence that tumour-/tissue-specific adhesion pathways can regulate the adhesion of TILs to tumour endothelium, suggesting that tissue-specific factors may regulate TIL recruitment to tumour in a manner analogous to that seen with normal T-cell recirculation (Salmi et al, 1992; Salmi et al, 1995; Yoong and Adams, 1996).

Thus, whether transfused TILs migrate back to sites of tumour or are 'lost' elsewhere will be determined by (1) the adhesion molecules expressed by the TILs and their state of activation; (2) the adhesion counter-receptors expressed by tumour endothelium; and (3) the adhesion counter-receptors on other host endothelium which would trap or recruit them elsewhere. We have undertaken an extensive phenotypic and functional analysis of TILs cultured from human melanoma to determine which adhesion molecules they express and use to bind endothelium in vitro. We have demonstrated that, compared with peripheral blood T cells, cultured TIL are highly activated with increased expression of several adhesion molecules and activation-dependent antigens. Furthermore, they bind avidly in vitro to both resting and cytokine-activated endothelial monolayers. These characteristics of cultured TILs are likely to have a profound influence on their ability to migrate to tumours in vivo.

### MATERIALS AND METHODS

### TIL preparations

TIL preparations from seven patients with malignant melanoma were studied. TILs were prepared as described previously (Yannelli, 1991). Briefly, tumour biopsies were removed from cancer patients in the Surgery Branch of the National Cancer Institute. Tumour tissue was cut into 1- to 3-mm$^3$ chunks and digested overnight in collagenase type IV (1 μg ml$^{-1}$), hyaluronidase (0.1 μg ml$^{-1}$) and DNAase (30 μg ml$^{-1}$). After digestion, the single-cell suspensions were passed through a sterile wire screen grid, washed three times in calcium- and magnesium-free Hanks' balanced salt solution (HBSS) and passed over Ficoll–Hypaque gradients to remove dead cells and red blood cells. The TIL cell cultures were established with $5.0 \times 10^5$ total cells ml$^{-1}$ in 24-well culture plates (Costar) in RPMI-1640 with 10% human AB serum (Bio-Whitaker, Walkersville, MD, USA) and antibiotics. This medium was mixed 1:1 (v/v) with AIM-V serum-free medium (Gibco, Grand Island, New York, NY, USA) and supplemented with 7200 IU ml$^{-1}$ IL-2 (Cetus Emeryville, CA, USA) and 10% (v/v) lymphokine-activated killer cell-conditioned medium prepared as previously described (Yannelli, 1991). TIL densities were maintained at $5.0 \times 10^5$ ml$^{-1}$ by splitting every 3–5 days with fresh medium containing IL-2. TILs used in the study had been in culture for between 30 and 45 days.

### Cells and culture reagents

Allostimulated T cells were used for comparison in the phenotypic studies. These cells were originally expanded for use in HLA typing by allostimulation with DP mismatched feeder cells. After 2 days, 15–20% T-cell growth factor (IL-2) was added to the cultures and the cells were then maintained in T-cell growth factor and restimulated with antigen every 7–10 days. Cells were cultured for a total of 24 days before being cryopreserved. The T-cell growth factor used was supernatant from 0.08% phytohaemagglutinin (PHA)-P activated peripheral blood lymphocytes that contained IL-2. These cells were chosen for comparison with TIL in the phenotypic studies because they had been stimulated with specific antigen and subsequently cultured in high doses of IL-2 and cryopreserved under similar conditions. However, there are obviously important differences between the two cell types, the allostimulated cells were CD4 T cells derived from peripheral blood, whereas TILs are usually CD8 and are derived from tissue.

Human peripheral blood T cells (PBTs) were isolated from normal donors by rigorous negative immunoselection with magnetic beads as previously described (Horgan and Shaw, 1991) using a cocktail of monoclonal antibodies (MAbs) against HLA class II on B cells, activated T cells and monocytes (IVA12), CD20 on B cells (1F5), CD16 on NK cells (3G8), CD11b on monocytes (NIH11b-1), CD14 on monocytes (MMA) and glycophorin on erythrocytes (10F7). Purity of the T cells was greater than 98%.

Human umbilical venous endothelial cells (HUVECs) were isolated and cultured as previously described (Shimizu et al, 1991*a*) in M199 media containing 20% fetal calf serum (FCS, Hyclone), 90 μg ml$^{-1}$ preservative-free porcine heparin, 20 μg ml$^{-1}$ endothelial cell growth supplement and antibiotics. All studies were done on confluent monolayers at passage 2 or less in 24-well plates (Costar).

The HMEC-1 dermal microvascular cell line was cultured as previously described (Ades et al, 1993) in EBM media (Clonetics) containing 10% FCS, 10 ng ml$^{-1}$ epidermal growth factor (EGF) and 100 ng ml$^{-1}$ hydrocortisone plus antibiotics. All studies were done using confluent monolayers in 48-well plates (Costar).

### Monoclonal antibodies

The antibodies used for the phenotypic studies are listed in Table 1. Monoclonal antibodies were used as ascites fluid, culture supernatant or purified antibody. All were used in saturating concentrations.

The following MAbs were used at 10 μg ml$^{-1}$ purified antibody to block adhesion: NIH11b-1 (CD11b/MAC-1 α-chain) (Horgan et al, 1990), MHM24 (CD11a/LFA-1 α-chain) (Hildreth et al, 1983), MHM23 (CD18/β2 integrin β-chain) (Hildreth et al, 1983), 2G7 (VCAM-1) (Graber et al, 1990), 7A9 (E-selectin) (Graber et al, 1990), NIH45-2 (CD45) (Shimizu et al, 1991*b*), MAb 84H10 (ICAM-1) (Makgoba et al, 1988), L25 (VLA-4 α-chain) (Clayberger et al, 1987; Takada et al, 1989), MAB-13 (Matsuyama et al, 1989) and 4B4 (Coulter Electronics, Hialeah, FL, USA) (CD29/VLA β-chain) (Matsuyama et al, 1989), MAb-16 (VLA-5 α-chain) (Matsuyama et al, 1989), Act-1 (α4β7) (Lazarovits et al, 1984), HML-1 ($\alpha^{IEL}\beta7$) (Schieferdecker et al, 1990).

### Flow cytometry

Cell surface phenotyping was done on TILs and PBTs as described previously (Schweighoffer et al, 1993). Approximately $1–2 \times 10^6$ cells were washed twice with FACS medium (HBSS containing 0.2% human serum albumin and 0.2% sodium azide), incubated with specific MAb at saturating concentrations for 30 min at 4°C, washed twice with medium, stained with goat anti-mouse or anti-rat fluorescein isothiocyanate (FITC) for 30 min at 4°C, washed twice and analysed on a modified Becton Dickinson FACS-II. Analysis was carried out using Reproman (TrueFacts Software, Seattle, WA, USA). Cryopreserved TILs were used for the studies as in cryopreserved T cells the L-selectin of the molecules that we looked at was markedly lower compared with fresh T cells (data not shown).

© Cancer Research Campaign 1997

**Table 1** Median channel fluorescence for the 55 antibodies studied on cultured TILs from seven patients, PBT from a healthy control and cultured human allostimulated T cells.

| Specificity | MAb | TILs (*n*=7) | | TIL (median) | Allostimulated T cells | PBTs |
|---|---|---|---|---|---|---|
| | | Mean | s.d. | | | |
| Control | 1.9.9 | 50 | 10 | 52 | 41 | 32 |
| CD1b | 100-1A5 | 65 | 8 | 62 | 116 | |
| CD2 | 49 | 137 | 15 | 141 | 172 | 117 |
| CD3 | UCHT1 | 135 | 25 | 145 | 158 | 152 |
| CD7 | 3A1 | 127 | 11 | 131 | 108 | 147 |
| CD8 | B9.8 | 132 | 44 | 143 | 72 | 41 |
| CD9 | 50H19 | 44 | 12 | 50 | 64 | 65 |
| CD11A | MHM24 | 143 | 16 | 137 | 161 | 137 |
| CD11B | NIH11b | 84 | 26 | 101 | 157 | 43 |
| CD11C | SHCL3 | 60 | 5 | 57 | 60 | 41 |
| CD15s | SNH3 | 66 | 19 | 68 | 27 | |
| CD18 | MHM23 | 141 | 13 | 148 | 170 | |
| CD21 | B2 | 32 | 8 | 32 | 37 | |
| CD25 | TAC | 67 | 23 | 55 | 139 | 32 |
| CD26 | TA1 | 136 | 3 | 136 | 127 | |
| CD27 | CLB2711 | 61 | 17 | 68 | 130 | |
| CD29 | 4B4 | 118 | 12 | 111 | 148 | |
| CD30 | BERH6 | 88 | 16 | 84 | 74 | 34 |
| CD31 | NIH31–2 | 70 | 24 | 79 | 34 | 96 |
| CD38 | OK10 | 44 | 15 | 44 | 149 | 88 |
| CD38 | WM32 | 58 | 12 | 57 | 57 | |
| CD39 | OKT28 | 129 | 16 | 127 | 137 | 30 |
| CD41 | UR-1663 | 45 | 5 | 45 | 58 | |
| CD43 | OTH71C5 | 129 | 18 | 134 | 176 | 173 |
| CD44 | NIH44 | 131 | 12 | 128 | 178 | 169 |
| CD45RA | G1.15 | 81 | 4 | 83 | 106 | |
| CD45RB | PD7 | 98 | 23 | 95 | 123 | |
| CD45RO | UCHL-1 | 144 | 9 | 149 | 204 | |
| CD49A | VLA-1 | 61 | 18 | 60 | 51 | 29 |
| CD49B | CLB-THR-4 | 105 | 11 | 105 | 134 | 48 |
| CD49C | PIB5 | 73 | 26 | 64 | 52 | 31 |
| CD49D | L25 | 99 | 14 | 102 | 141 | 86 |
| CD49E | P1D6 | 60 | 10 | 62 | 74 | 50 |
| CD49F | GOH3 | 48 | 7 | 49 | 48 | 69 |
| CD49F | DC5-6 | 61 | 19 | 53 | 72 | |
| CD54 | 84H10 | 86 | 6 | 85 | 133 | 63 |
| CD55 | F2B72 | 105 | 14 | 112 | 122 | |
| CD58 | TS2/9 | 90 | 19 | 98 | 123 | 43 |
| CD60 | UM4D4 | 50 | 5 | 53 | 41 | |
| CD61 | CLBthr-1 | 45 | 4 | 44 | 37 | |
| CD62L | LEU8 | 60 | 15 | 62 | 52 | 134 |
| CD65 | VIM-8 | 42 | 6 | 39 | 33 | |
| CD69 | L78 | 73 | 14 | 79 | 39 | 34 |
| CD70 | KI24 | 110 | 13 | 112 | 107 | |
| CD71 | L5.1 | 45 | 7 | 45 | 85 | |
| CD73 | AD-2 | 46 | 7 | 48 | 37 | |
| CD75 | LN-1 | 65 | 31 | 50 | 28 | |
| CD76 | CRIS-4 | 50 | 9 | 51 | 39 | |
| CD99 | TU12 | 111 | 48 | 82 | 171 | 75 |
| CD103 | HML-1 | 71 | 27 | 65 | 120 | 29 |
| CDw109 | LDA-1 | 69 | 16 | 70 | 75 | |
| CD122 | MIKB2 | 86 | 11 | 86 | 130 | |
| *Unclustered antigens* | | | | | | |
| CLA | HECA-452 | 82 | 24 | 75 | 31 | 31 |
| Alpha4beta7 | ACT-1 | 66 | 12 | 57 | 113 | |
| Class I | W6/32 | 189 | 5 | 184 | 197 | |
| Class II | IVA12 | 169 | 29 | 182 | 201 | |

The mean, s.d. and the median values for the seven TIL are shown. CD4 is omitted because only one of the seven TIL preparations consisted of $CD4^+$ TIL.

### Cell adhesion assays

*Binding to endothelium*

Binding of TILs and PBT to HUVECs was assessed as previously described (Shimizu et al, 1991*a*). Briefly, HUVECs were plated onto gelatin-precoated 24-well plates (Costar) and cultured for 48 h until confluent, activated by exposure to 1 ng ml$^{-1}$ IL-1β in medium (RPMI/10% FCS) for 4 h at 37°C and then washed twice with medium immediately before addition of T cells. TILs or PBTs were labelled with $^{51}$Cr, and 300 000 T cells were added to each well in a final volume of 300 μl. In order to assess adhesion of acutely activated T cells, cells were preactivated by incubation for 20 min with 10 ng ml$^{-1}$ PMA (Sigma Chemical, St Louis, MO, USA) before washing and addition to the HUVEC monolayer. When blocking by MAbs was assessed, binding was carried out in the continuous presence of antibody; all MAbs were used at a saturating concentration of 10 μg ml$^{-1}$, which has been shown in previous studies to maximally inhibit the relevant adhesive interaction (Graber et al, 1990; Shimizu et al, 1990*a*, *b*, *c*; van Seventer et al, 1990). Plates were incubated for 30 min at 37°C and then gently washed twice with RPMI/10% FCS media at room temperature to remove non-adherent T cells. Contents of each well containing adherent T cells were lysed with 300 μl of 1% Triton X-100 and γ-emissions were counted. Data are expressed as mean per cent of cells binding [(counts from wells/counts from 300 000 lysed cells) × 100]. Because the differences observed between freshly isolated TIL and TIL that had been cryopreserved were small, cryopreserved TIL were used for most of the studies. Binding to HMEC-1 was assessed in a similar assay, except that cells were grown to confluence in EBM and then in RPMI/10% FCS for the final 18 h during which activation was done using 50 units ml$^{-1}$ TNF-α which has previously been shown to induce optimal activation of HMEC-1 (Swerlick et al, 1992).

*Actin polymerization*

The morphology of TILs and PBTs and the distribution of polymerized filamentous actin (F-actin) were assessed by rhodamine phalloidin staining and laser scanning confocal microscopy. Cells were settled for at least 60 min at 37°C on plastic slides that had been coated with 20 μg ml$^{-1}$ fibronectin. The cells were then fixed with 3.7% formaldehyde for at least 1 h at 4°C, permeabilized by incubating with 2 mg ml$^{-1}$ lysophosphatidyl choline (LPC) (Sigma) and total F-actin detected by staining with 300 units rhodamine-phalloidin for 30 min at 4°C. Cells that were fixed and stained with rhodamine-phalloidin were analysed with a Nikon Microphot-FX microscope (plan 40× objective lens) connected to a BioRad MRC 600 laser scanning confocal microscope (BioRad Life Sciences Group, Melville, NY, USA).

### Statistics

Comparisons between groups were analysed using the Mann–Whitney *U*-test for non-parametric data. A level of 2*P*<0.05 was taken as significant. Trends were analysed using Kruskal–Wallis one-way ANOVA.

### RESULTS

### TIL have an abnormal highly activated morphology and cell surface phenotype

All the TIL preparations studied were T cells (> 95% CD3 positive); six were predominantly CD8$^+$ and one predominantly CD4$^+$. Although cultured TILs are T cells, they are markedly different from resting PBTs, with respect to both overall morphology and cell-surface phenotype. TILs are larger, more irregular and have increased foci of filamentous actin in the cytoskeleton, consistent with an activated state (Figure 1). TILs also have a markedly different phenotype from PBTs with respect to the levels of expression of cell-surface markers. We initially screened TIL preparations using 57 MAb reactive with 54 different T-cell surface molecules and compared them with activated cultured T cells (SB6) (using all MAbs) and with PBTs (using 29 of the MAbs). We have highlighted data obtained with 27 of these MAbs in Figure 2 (PBTs compared with TILs) and 35 in Figure 3 (alloactivated T cells compared with TILs). The selection of the MAb was based on several criteria: (1) marked differences in expression between TIL and SB6 or PBT; (2) heterogeneity of expression on

A B

**Figure 1** TILs are larger, more polarized and have increased filamentous actin compared with resting peripheral blood T cells. The figure shows rhodamine-phalloidin staining of filamentous actin in (**A**) TILs and (**B**) resting PBTs settled on fibronectin-coated slides. Images were collected with a Nikon Microphot-FX microscope (plan 40× objective lens) connected to a BioRad MRC 600 laser scanning confocal microscope

© Cancer Research Campaign 1997

Figure 2 TILs have higher levels of many molecules than PBTs, consistent with their activated state. This scatterplot shows the median channel fluorescence of 27 antibodies to cell surface markers shown on TILs (median of the seven preparations) compared with the median channel fluorescence of each antibody on PBTs. The name of the molecule is positioned at the appropriate co-ordinates. A solid line marks the position for molecules expressed at equivalent levels on TILs and PBTs. The fine dotted lines represent the staining for each cell type with 1.9.9, an irrelevant mouse antibody. In all cases the background staining with 1.9.9 was higher on TILs than on PBTs (Table 1)

Figure 3 TILs have higher levels of several potentially important molecules than alloantigen-stimulated T cells. The median value of the seven TILs preparations for median channel fluorescence of 35 antibodies to cell surface markers is shown and compared with the same antibodies on allostimulated T cells (human T cells activated by alloantigen and cultured under similar conditions to TILs). The fine dotted lines represent the staining for each cell type with 1.9.9, an irrelevant mouse antibody

TIL; or (3) probable relevance to migration. The excluded MAb were generally either negative on TIL or expressed at roughly equivalent levels on TIL and the other cell preps. Table 1 provides a comprehensive tabulation of all the studies.

### The cell-surface phenotype of TILs is very different from that of peripheral blood T cells

The cell-surface antigens studied can be divided into three groups based on their staining patterns on TILs compared with PBTs (Figure 2):

1. those that were expressed at least twofold higher on TILs than on PBTs, comprising five integrins (CD49a, CD49b, CD49c, CD11b, CD103), two additional adhesion molecules (CLA and CD58) and four activation markers (HLA class II, CD30, CD39, CD69);
2. those with higher expression on PBTs, including three adhesion molecules (CD43, CD44 and CD62L) and an activation/signalling molecule (CD38);
3. the rest, showing smaller differences between PBTs and TILs or being expressed at similar levels.

Thus, there are marked differences between TILs and PBTs with respect to nine different adhesion molecules, as well as five activation markers.

### Is the phenotype of the TILs simply a function of activation during in vitro culture?

To answer this question we compared TILs with a preparation of allostimulated T cells that had been expanded in vitro for approximately the same time and with similar IL-2 supplementation (Figure 3). The allostimulated cells were predominantly CD4; however previous studies have shown that CD4 and CD8 T cells are similar in their surface phenotype with respect to many markers (Shaw et al, 1994). While we accept that these cells are not an ideal cell type for comparison with the TILs, we believe that they do provide useful information about the regulation of the markers studied. Many of the molecules were expressed at similar levels on both TILs and allostimulated T cells, reflecting similarities in culture and activation between the two T-cell preparations. However, marked differences were observed for other markers. Firstly, three molecules were expressed at twofold higher levels on TILs than allostimulated T cells: (a) the cutaneous lymphocyte-associated antigen, CLA; (b) CD69, an acute activation marker; and (c) CD31, an adhesion-inducing molecule (Tanaka et al, 1992). Secondly, 12 molecules were expressed at higher levels on allostimulated T cells than on TIL: (a) the integrins $\alpha 4\beta 7$, $\alpha IEL\beta 7$, CD49b, CD49d, and CD11b; (b) the adhesion molecules CD99 (E2), CD43, CD44 and CD54; and (c) activation antigens CD25, CD38 (OKT10) and CD71.

### Adhesion molecules show heterogeneity of expression within and between TIL preparations

Median channel fluorescence provides a statistical summary of complex data that conceal heterogeneity both within a given cell preparation and between preparations. Both kinds of heterogeneity are illustrated in Figure 4. One of the least heterogeneous molecules is the integrin LFA-1; although median fluorescence is consistent between the three profiles, there is marked heterogeneity within one of the TIL preparations, demonstrating that even LFA-1 is differentially regulated on different cells. Such

© Cancer Research Campaign 1997

**Figure 4** Histograms of selected molecules comparing expression on TIL with PBT. Each panel shows staining for PBT (dashed line) and two TIL preparations: the two TIL preparations shown for a marker are ones whose median channel fluorescence is either side of the median TIL for all seven patients heterogeneity was seen with most of the markers. Heterogeneity between TIL is illustrated by VLA-4. Even these two TILs that are close to the 'median TIL' differ substantially from each other. Because of the averaging process, VLA-4 does not appear to be remarkable in Figure 2. As data are collected on a four-decade log scale, a twofold difference corresponds to about 20 channels. Thus, the TIL shown differ more than 10-fold in median expression of VLA-4.

Among the other adhesion molecules, we illustrate two that are increased on TILs (CLA and HML-1) and two that are reduced several-fold compared with PBTs. Among the traditional 'activation markers' (CD69, HLA class II, CD30, CD38), three are increased but one is decreased from its low basal expression on resting T cells (CD38).

### TILs display strong activation-independent binding to endothelium

We investigated the capacity of TILs to bind to endothelium in standard in vitro assay of leucocyte binding to cultured human umbilical vein endothelial cells (HUVECs) (Figures 5 and 6) and a transformed skin-derived microvascular endothelial cell line (HMEC-1) (Figure 6). TILs bound avidly. More than 50% of TILs bound to resting endothelium, whereas only 10–15% of resting PBTs bound. Activation of TILs by phorbol ester did not cause a further increase in binding, suggesting that integrins on TILs are already fully activated. This contrasts with the marked increase in PBT adhesion to both resting and activated endothelium after phorbol ester stimulation (Figure 5).

© Cancer Research Campaign 1997

**Figure 5** TIL binding to HUVEC is increased by activation of the endothelium but not by activation of TILs. The bars show the per cent cells binding to HUVEC monolayers in a representative experiment. The solid bars represent PBT from a healthy donor and the hatched bars and open bars two melanoma-derived cultured TIL preparations. The symbols on the left margin indicate whether the lymphocytes were activated by PMA (10 ng ml$^{-1}$) or the HUVECs activated by exposure to IL-1 1 ng ml$^{-1}$ for 6 h. The binding of PBTs is low when both cell types are resting and is increased by activation of both cell types. TILs, on the other hand, show high binding without activation that is increased by activating the endothelium but unaffected by phorbol ester activation of TILs

**Figure 6** TIL bind to two different endothelial cell monolayers, HUVEC and HMEC-1, in a similar manner. (**A**) Binding of TILs to resting HMEC-1 can be partly blocked using antibodies to CD18, the β2 chain of LFA-1 (MHM23). Inhibition is enhanced by adding antibodies to CD29, the β1 chain of VLA-4 (MAb13). The pathways of TIL binding are similar to those of PMA-activated PBTs. Resting PBTs show little binding. The bars represent mean results ± s.e.m. from seven TILs and mean results of triplicate assay of one representative PBT. (**B**) Binding of TILs to activated HMEC-1 was only minimally inhibited by CD29 antibodies alone but was significantly reduced when anti-CD18 MAbs were used and particularly when the antibodies were used together. The bars represent mean results ± s.e.m. from seven TIL preparations and mean results of triplicate assay of one representative PBT. (**C**) Binding of TILs to HUVEC monolayers showed a similar hierarchy of pathways with the exception that MAb to E-selectin (7A9) had a greater effect on activated HUVEC than on activated HMEC-1. This reflects the low levels of E-selectin expressed by activated HMEC-1

### TILs bind to endothelium via activated integrin-mediated adhesion

Combinations of blocking MAb were used to define the contribution of the three main adhesion pathways (Figure 6):

1. β1 integrin (VLA-4) on TILs to VCAM-1 on endothelium;
2. β2 integrin (LFA-1) on TILs to ICAM-1 or ICAM-2 on endothelium;
3. E-selectin on endothelium to its carbohydrate ligand on TILs (CLA and/or another carbohydrate receptor).

The blocking antibodies were used either alone or in combinations of two and three antibodies to allow the contribution of each pathway to be assessed as described previously (Shimizu et al, 1991*a*).

TILs binding to resting endothelium involved β2 integrins and to a lesser extent β1 integrins (Figure 6A). Adhesion was blocked substantially by MAb against the β2 integrin chain (Mann–Whitney *U*-test, $2P < 0.015$), presumably by blocking LFA-1 to ICAM-1 and ICAM-2. In contrast, MAb against β1 integrins alone had a minimal effect ($2P = 0.456$). The use of the two antibodies in combination to block both β1 and β2 integrins caused a fall in binding compared with the use of the anti-β2 antibody alone ($2P < 0.05$), confirming that β1 integrins are also involved, although their contribution is masked by the dominant binding via β2 integrins. Binding to resting HUVECs was similar to that for HMEC-1 (Figure 6A). E-selectin is not expressed on resting endothelium and was therefore not involved.

Binding to activated endothelium involves integrins and selectin-mediated pathways. Activation of endothelium by exposure to either IL-1 (HUVEC) or TNF-α (HMEC-1) increased the percentage of TILs that bound (Figure 6B vs 6A). As seen with resting endothelium, binding to activated endothelium involved both β1 and β2 integrins, with the β2 integrin pathway dominating (β1 integrin compared with control, $2P = 0.07$; β2 integrin, $2P < 0.001$; β1+β2, $2P < 0.0006$; β1+β2 compared with β2 alone, $2P < 0.002$). E-selectin MAb, by itself, had no effect on binding; however, addition of E-selectin MAb to the mix of β1/β2 MAb caused a small but definite reduction in TIL binding to HUVECs compared with β1/β2 mix alone but had no effect when HMEC-1 endothelial cells were used, which is consistent with the very low levels of E-selectin expressed on HMEC-1 in our culture system (data not shown). Overall, the pattern of adhesion inhibition for unactivated TILs is similar to the pattern with PMA-activated PBTs.

© Cancer Research Campaign 1997

### DISCUSSION

The goal of adoptive immunotherapy is to reconstitute patients with cultured TILs that subsequently migrate back to tumour deposits and generate an anti-tumour response (Rosenberg et al, 1986). The therapeutic efficacy of TILs will depend on two interrelated factors: their ability to mount an effective anti-tumour response on encountering tumour cells (Itoh et al, 1986; Spiess et al, 1987; Aebersold et al, 1990; Yannelli, 1991) and, equally important, their ability to reach tumour sites in sufficient numbers to have a clinically significant effect. Relatively little is known about the factors that regulate TIL recruitment to tumour sites, despite the fact that such factors will be crucial for the clinical efficacy of TILs (Whiteside and Herberman, 1992).

Before cultured TILs can migrate from the circulation into tissue they must first recognize and bind to tumour endothelium, a process determined by the adhesion molecules expressed on TILs and the presence of appropriate ligands on tumour endothelium (Butcher, 1991; Shimizu et al, 1992; Whiteside and Herberman, 1992). In the present study we determined the detailed phenotype of cultured TILs, with particular reference to the adhesion molecules they express, and assessed their ability to bind to endothelium in vitro. We found gross differences between TILs and normal circulating T cells that are likely to have a profound effect on the migratory behaviour of infused TIL in vivo.

Cultured TILs showed enhanced binding to both resting and activated endothelium that was mediated largely by LFA-1 and VLA-4, the most important T-cell integrins in endothelial-binding (Shimizu et al, 1991*a*, 1992). Furthermore, treatment of TILs with phorbol ester did not increase binding, suggesting that integrins on TILs are fully activated. This is in marked contrast to the low levels of integrin-mediated binding seen with resting peripheral blood T cells in the absence of activation (Figure 5; Shimizu et al, 1991*a*). This activation-independent adhesion is not merely a reflection of increased surface expression of integrins on TIL (Figure 2), but rather of an altered functional state of those integrins.

What are the potential implications of these findings for TILs homing in vivo? Increased integrin activation on cultured TILs will disrupt the normal regulation of endothelial binding. On the one hand, this might increase the numbers of TILs binding to tumour endothelium and thereby increase the numbers entering tumour tissue. However, the specificity of endothelial binding will be lost, with the consequence that TILs can bind to multiple vascular beds as well as to tumour endothelium with a consequent reduction in the specificity of TIL migration. In particular, this is likely to occur in the sinusoids of the liver and spleen and in the small vessels of the lung where cells come into intimate contact with endothelium that constitutively expresses ICAM-1 and, in the case of the lung and liver, low levels of VCAM-1 (Rice et al, 1991; Adams et al, 1994; Dunkley et al, 1995). The larger, more irregular shape of TILs (Figure 1; Whiteside and Herberman, 1992) will reduce their deformability and lead to increased contact between circulating TIL and the wall of small vessels, thereby allowing time for the activated integrins to engage these endothelial ligands (Adams and Nash, 1996). This is particularly likely to happen in low-flow systems, such as the hepatic and splenic sinusoids (Adams, 1996). In addition, the large size of TILs will result in physical trapping in the small-calibre vessels of organs such as the lung (Adams and Nash, 1996).

These concerns are supported by clinical studies in which indium-labelled TILs were infused into patients and their in vivo distribution subsequently assessed by scanning with a gamma-camera. The vast majority of the infused cells were detected in the liver, spleen and lung within minutes of infusion, and only a small proportion reached tumour deposits (Poppema et al, 1983; Griffith et al, 1989). This inefficient trafficking of cultured TILs is in contrast to the efficient removal of relevant T cells from the circulation that occurs in normal homing (Fisher and Ottaway, 1990; Picker and Butcher, 1992). Thus, it appears that cultured TILs have an impaired ability to migrate selectively to their intended tissue target. This explains why large numbers of cultured TILs must be infused to insure that at least some reach the tumour.

In addition to the size and activation status of cultured TILs, their altered cell-surface phenotype is likely to have a profound effect on their migration in vivo. In the present study we report phenotypic data on a large number of molecules, some of which we predict will have a direct effect on TIL migration. The significance of others is likely to become apparent as our understanding of them increases.

One molecule of great potential relevance for homing to melanoma, a skin-derived cancer, is the cutaneous lymphocyte antigen (CLA), which is a ligand for E-selectin (Picker et al, 1990*a*). CLA, a heavily glycosylated carbohydrate epitope on cell surface glycoproteins, is expressed at low levels on less than 20% of PBT (Berg et al, 1991; Picker et al, 1993*a*). In vivo, CLA is expressed on cutaneous lymphomas (Picker et al, 1990*a*) and on 85% of T cells at sites of skin inflammation (Picker et al, 1990*a*). In contrast, it is detected on less than 5% of T cells in inflamed non-cutaneous sites (Adams et al, 1996). CLA has therefore been proposed as a skin homing receptor (Picker and Butcher, 1992). As we found high levels of expression of CLA on most of the TIL preparations and as melanoma endothelium expresses the CLA receptor E-selectin (Rohde et al, 1992), the CLA–E-selectin interaction may facilitate TIL migration into melanoma. The fact that we did not see a major contribution from E-selectin in the adhesion assays may be because E-selectin/CLA mediates the initial tethering adhesion that leads to rolling of the T cell on the vessel wall. Static adhesion assays do not reflect the significance of this step because integrin binding dominates in such systems (Spertini et al, 1991). Thus, the small effect we saw with anti-E-selectin is unlikely to be a true reflection of its contribution in vivo. The presence of CLA on TILs would promote binding to E-selectin on tumour endothelium in vivo, resulting in tethering – the first step in the adhesion cascade. The usual requirement for a second step to trigger integrin activation would be bypassed because of the preactivated status of TIL integrins, allowing them to engage their ligands, ICAM-1 and VCAM-1, on tumour endothelium (Rohde et al, 1992). LFA-1 might be expected to dominate as ICAM-1 is expressed on tumour endothelium at far greater levels than VCAM-1 (Rohde et al, 1992).

From our studies it would appear that CLA expression is a characteristic of these TIL rather than an effect of their culture conditions. The findings that support this conclusion are:

1. the expression of CLA does not increase on peripheral blood T cells activated with mitogens in vitro (Picker et al, 1993*a*);
2. CLA was one of only three antigens that showed increased expression on TIL compared with allostimulated T cells;
3. TILs may have a homing 'phenotype' characteristic of skin, as TILs also expressed lower levels of the gut homing receptors $\alpha IEL\beta 7$ and $\alpha 4\beta 7$ (Picker et al, 1990*b*; Schweighoffer et al, 1993) compared with alloantigen-stimulated T cells;

© Cancer Research Campaign 1997

4. CLA is not expressed by TILs derived from either primary hepatic tumours or primary or secondary colonic carcinoma (Yoong and Adams, unpublished observations; Yoong and Adams, 1996; Yoong et al, 1996).

Thus, the CLA-high/LFA-1-high/α4β7-low/αIELβ7-low phenotype of TILs would be predicted to favour homing to skin tissues (Picker and Butcher, 1992; Picker et al, 1993*a*; Schweighoffer et al, 1993). If the highly activated state of LFA-1 and VLA-4 on TILs could be reduced to prevent non-specific adhesion to other vascular beds, this tissue-specific homing phenotype should result in efficient recruitment of infused TILs to tumour. The endothelial cell line we used in the adhesion studies, HMEC-1, is derived from microvascular endothelium in the skin. However, this cell line may differ substantially from the endothelium within melanoma tumour tissue in vivo and, in the long run, it will be important to understand details of interactions of TILs with various specialized kinds of endothelium, including endothelium in tumour vessels.

Other molecules with potential roles in endothelial binding that were detected on TILs included CD31, CD43 and CD44. CD31, a member of the immunoglobulin superfamily, has a unique distribution on peripheral blood T cells, being expressed on all naive CD8 T cells, 50% of CD8 memory cells and 50% of CD4 naive cells. CD31 expression was heterogeneous both within and between TIL preparations. The CD4 TIL preparation studied was largely negative, as might be expected from the distribution of CD31 in peripheral blood T cells. Although there was variation in the expression of CD31 between different CD8 TIL preparations, all of them contained a substantial subset of cells that were CD31 positive (Table 1), and it is possible that CD31 expression might have facilitated the original entry of this TILs into tumour. This is supported by the proposal that CD31 on T cells can act as an amplifier of T-cell integrin function when it engages an, as yet unknown, endothelial receptor (Shimizu et al, 1992; Tanaka et al, 1992) and by recent studies proposing a role for CD31 in the entry of TILs into murine tumours (Schmitt-Verhulst, 1994) (B Imhof, personal communication). However, CD31 expression cannot be an absolute requirement for TIL recruitment as the CD31-dull $CD4^+$ TILs that we studied (TIL6) migrated to tumour deposits in vivo.

Both CD43 and CD44 were reduced on TILs compared with PBTs. CD44 is an abundant multifunctional cell surface molecule that was initially inferred to be a tissue-specific homing receptor (Jalkanen et al, 1987). However, because of its broad tissue distribution it may have a more general role in facilitating binding to endothelium via its ligand hyaluronate (Aruffo et al, 1990). Decreased CD44 on TILs may therefore reduce efficiency of endothelial binding. In contrast, reduced expression of CD43, the predominant cell surface mucin on lymphocytes (*l*) on TILs might be expected to increase endothelial adhesion (McLean, 1994). The inference that mucins maintain a repulsive barrier around the cell is based on findings with CD43 (Ardman et al, 1992; Manjunath et al, 1993). For example, targeted disruption of the CD43 gene in the T-cell line CEM enhances homotypic adhesion and binding to fibronectin (Manjunath et al, 1993).

In addition to the 'adhesion molecules' discussed above, TILs also differ from PBTs in their expression of 'activation markers'. Most of these activation markers are expressed more on TILs than on PBTs (e.g. CD30, CD39, CD69, HLA class II). An exception is CD38, usually considered to be an activation marker, which is expressed at lower levels on TILs than on PBTs. These molecules may be important for TIL function. For instance, CD69 is a C-type lectin (Lopez-Cabera et al, 1993) likely to contribute to adhesion and CD38 has been implicated in binding to endothelium (Xu et al, 1994). Furthermore, both CD69 and CD38 are involved in signal transduction and cell activation (Nakamura et al, 1989; Moretta et al, 1991; Tugores et al, 1992; Xu et al, 1994) and CD30, a member of the TNF receptor family, could modulate TIL effector function by interacting with the CD30 ligand in the tumour (Faustman et al, 1982).

Although all these molecules are expressed with cellular activation, they show different regulation with respect to which subsets of cells express them, which signals induce them and the kinetics of their expression (Crabtree, 1989). This is illustrated by the contrast between TILs and alloactivated T cells (Figure 3); CD69 and CD30 are expressed at higher levels on TILs, whereas CD25 and CD38 are expressed at higher levels on allostimulated T cells. Thus, the detailed phenotype of these activated T cells is regulated in a complex manner that depends in part on the original character of the activated cell as well as on the exact details/timing of culture.

The fact that the phenotype of TILs is influenced by the details of their culture implies that it is susceptible to modification. As the physiological homing of T cells is highly selective and exquisitely regulated, the generation of TILs with a more physiological phenotype might result in restoration of their specific homing potential. The aim of such manipulation would be to maintain expression of molecules that are unique to TIL, such as the skin homing receptor CLA, while reducing the non-specific activation of, for instance, integrins, thereby allowing the original character of the TILs to emerge. Theoretically, such a strategy could produce TILs that show reduced binding to endothelium in normal tissues but maintain their ability to selectively bind tumour endothelium, thereby increasing delivery to tumour sites. These observations have wider implications with the increasing use of cultured leucocytes and adoptive immunotherapy in other clinical situations.

### ACKNOWLEDGEMENTS

We thank the staff of the Clinical Immunotherapy Laboratory, Surgery Branch, NCI, for their help in preparing TIL samples; Liana Harvath for help with the confocal microscopy of actin staining; Yoshiya Tanaka and Tamas Schweighoffer for helpful advice; Dr Louis Picker for critical review of the manuscript; many generous colleagues for providing MAb; G Ginther-Luce and N Graber for technical assistance; our volunteer blood donors and the NIH Blood Bank. DHA was supported by the Eli Lilly/Medical Research Council 1991/1992 Travelling Fellowship and the Fogarty Exchange Program.

### REFERENCES

Adams DH (1996) Lymphocyte-endothelial interactions in hepatic inflammation. *Hepatogastroenterology* **43**: 32–43

Adams DH and Shaw S (1994) Leucocyte endothelial interactions and regulation of leucocyte migration. *Lancet* **343**: 831–836

Adams DH and Nash GB (1996) Disturbance of leukocyte circulation and adhesion to endothelium as factors in circulatory pathology. *Br J Anaesth* **77**: 17–31

Adams DH, Burra P, Hubscher SG, Elias E and Newman W (1994) Endothelial activation and circulating vascular adhesion molecules in alcoholic liver disease. *Hepatology* **19**: 588–594

Adams DH, Williams A, Hubscher SG and Robinson M (1996) Expression of E-selectin (CD62E) and E-selectin ligands in human liver inflammation. *Hepatology* **24**: 533–538

Ades EW, Candal FJ, Swerlick RA, George VG, Summers S, Bosse DC and Lawley TJ (1993) HMEC-1: establishment of an immortalized human microvascular endothelial cell line. *J Invest Dermatol* **99**: 683–690

Aebersold P, Hyatt C, Johnson S, Hines K, Korcak L, Sanders M, Lotze M, Topalian S, Yang J and Rosenberg SA (1991) Lysis of autologus tumor cells by tumor-infiltrating lymphocytes correlates with clinical response in patients with malignant melanoma. *J Natl Cancer Inst* **83**: 932–937

Ardman B, Sikorski MA and Staunton DE (1992) CD43 interferes with T-lymphocyte adhesion. *Proc Natl Acad Sci USA* **89**: 5001–5005

Aruffo A, Stamenkovic I, Melnick M, Underhill CB and Seed B (1990) CD44 is the principal cell surface receptor for hyaluronate. *Cell* **61**: 1303–1313

Berg EL, Yoshino T, Rott LS, Robinson MK, Warnock RA, Kishimoto TK, Picker LJ and Butcher EC (1991) The cutaneous lymphocyte antigen is a skin lymphocyte homing receptor for the vascular lectin endothelial cell-leukocyte adhesion molecule 1. *J Exp Med* **174**: 1461–1466

Butcher EC (1991) Leukocyte–endothelial cell recognition: three (or more) steps to specificity and diversity. *Cell* **67**: 1033–1036

Butcher EC and Picker LJ (1996) Lymphocyte homing and homeostasis. *Science* **272**: 60–66

Clayberger C, Krensky AM, McIntyre BW, Koller TD, Parham P, Brodsky F, Linn DJ and Evans EL (1987) Identification and characterization of two novel lymphocyte function-associated antigens, L24 and L25. *J Immunol* **138**: 1510–1514

Crabtree GR (1989) Contingent genetic regulatory events in T lymphocyte activation. *Science* **243**: 355–361

Dunkley M, Pabst R and Cripps A (1995) An important role for intestinally derived T cells in respiratory defense. *Immunol Today* **16**: 231–236

Faustman D, Lacy P, Davie J and Hauptfeld V (1982) Prevention of allograft rejection by immunization with donor blood depleted of Ia-bearing cells. *Science* **217**: 157–158

Fisher LL and Ottaway CA (1990) The kinetics of migration of murine CD4 and CD8 lymphocytes in vivo. *Reg Immunol* **3**: 156–162

Graber N, Gopal TV, Wilson D, Beall LD, Polte T and Newman W (1990) T-cells bind to cytokine-activated endothelial cells via a novel, inducible sialoglycoprotein and ELAM-1. *J Immunol* **145**: 819–830

Griffith KD, Read EJ, Carrasquillo JA, Carter CS, Yang JC, Fisher B, Aebersold P, Packard BS, Yu MY and Rosenberg SA (1989) In vivo distribution of adoptively transferred indium-111-labeled tumor infiltrating lymphocytes and peripheral blood lymphocytes in patients with metastatic melanoma. *J Natl Cancer Inst* **81**: 1709–1717

Hildreth JE, Gotch FM, Hildreth PD and McMichael AJ (1983) A human lymphocyte-associated antigen involved in cell-mediated lympholysis. *Eur J Immunol* **13**: 202–208

Horgan KJ and Shaw S (1991) Immunomagnetic purification of T cell subpopulations. Section 7.4. In *Current Protocols in Immunology*, Coligan JE, Kruisbeek AM, Margulies DH, Shevach EM and Strober W. (eds), pp. 7.4.1–7.4.5. Wiley Interscience: New York

Horgan KJ, Van Seventer GA, Shimizu Y and Shaw S (1990) Hyporesponsiveness of naive (CD45RA+) human T cells to multiple receptor-mediated stimuli but augmentation of responses by costimuli. *Eur J Immunol* **20**: 1111–1118

Hynes RO (1992) Integrins: versatility, modulation, and signaling in cell adhesion. *Cell* **69**: 11–25

Itoh K, Tilden AB and Balch CM (1986) Interleukin-2 activation of cytotoxic T lymphocytes infiltrating into human metastatic melanomas. *Cancer Res* **46**: 3011–3017

Jalkanen S, Bargatze RF, De Los Toyos J and Butcher EC (1987) Lymphocyte recognition of high endothelium: antibodies to distinct epitopes of an 85–95 kD glycoprotein antigen differentially inhibit lymphocyte binding to lymph node, mucosal, or synovial endothelial cells. *J Cell Biol* **105**: 983–990

Lazarovits AI, Moscicki RA, Kurnick JT, Camerini D, Bhan AK, Baird LG, Erikson M and Colvin RB (1984) Lymphocyte activation antigens. I. A monoclonal antibody, Act-I, defines a new late lymphocyte activation antigen. *J Immunol* **133**: 1857–1862

Lopez-Cabera M, Santis AG, Fernandez-Ruiz E, Blacher R, Esch F, Sanchez-Mateos P and Sanchez-Madrid F (1993) Molecular cloning, expression, and chromosomal localization of the human earliest lymphocyte activation antigen AIM/CD69, a new member or the C-type animal lectin superfamily of signal transmitting receptors. *J Exp Med*

Makgoba MW, Sanders ME, Luce GEG, Dustin ML, Springer TA, Clark EA, Mannoni P and Shaw S (1988) ICAM-1 a ligand for LFA-1-dependent adhesion of B, T and myeloid cells. *Nature* **331**: 86–88

Manjunath N, Johnson RS, Staunton DE, Pasqualini R and Ardman B (1993) Targeted disruption of CD43 gene enhances T lymphocyte adhesion. *J Immunol* **151**: 1528–1534

Matsuyama T, Yamada A, Kay J, Yamada KM, Akiyama SK, Schlossman SF and Morimoto C (1989) Activation of CD4 cells by fibronectin and anti-CD3 antibody. A synergistic effect mediated by the VLA-5 fibronectin receptor complex. *J Exp Med* **170**: 1133–1148

Moretta A, Poggi A, Pende D, Tripodi G, Orengo AM, Pella N, Augugliaro R, Bottino C, Ciccone E and Moretta L (1991) CD69-mediated pathway of lymphocyte activation: anti-CD69 monoclonal antibodies trigger the cytolytic activity of different lymphoid effector cells with the exception of cytolytic T lymphocytes expressing T cell receptor a/b. *J Exp Med* **174**: 1393–1398

Nakamura S, Sung S-SJ, Bjorndahl JM and Fu SM (1989) Human T cell activation. IV. T cell activation and proliferation via the early activation antigen EA1. *J Exp Med* **169**: 677–689

Picker LJ and Butcher EC (1992) Physiologic and molecular mechanisms of lymphocyte homing. *Annu Rev Immunol* **10**: 561

Picker LJ, Michie SA, Rott LS and Butcher EC (1990*a*) A unique phenotype of skin-associated lymphocytes in humans preferential expression of the HECA-452 epitope by benign and malignant T cells at cutaneous sites. *Am J Pathol* **136**: 1053–1068

Picker LJ, Terstappen LWMM, Rott LS, Streeter PR, Stein H and Butcher EC (1990*b*) Differential expression of homing-associated adhesion molecules by T cell subsets in man. *J Immunol* **145**: 3247–3255

Picker LJ, Treer JR, Ferguson-Darnell B, Collins PA, Bergstresser PR and Terstappen LW (1993*a*) Control of lymphocyte recirculation in man. II. Differential regulation of the cutaneous lymphocyte-associated antigen, a tissue-selective homing receptor for skin-homing T cells. *J Immunol* **1500**: 1122–1136

Picker LJ, Treer JR, Ferguson-Darnell B, Collins PA, Buck D and Terstappen LW (1993*b*) Control of lymphocyte recirculation in man. I. Differential regulation of the peripheral lymph node homing receptor L-selectin on T cells during the virgin to memory cell transition. *J Immunol* **150**: 1105–1121

Pockaj BA, Sherry R, Wei J, Yannelli JR, Carter CS, Leitman SF, Carrasquillo JR, White DE, Steinberg SM, Rosenberg SA and Yang JC (1994) Localisation of Indium-labelled tumor infiltrating lymphocytes to tumor in patients receiving adoptive immunotherapy: augmentation with cyclophosphamide and association with response. *Cancer* **73**: 1731–1737

Poppema S, Brocker EB, De Leij L, Terbrack D, Visscher T, Ter Haar A, Macher E, The TH and Sorg C (1983) In situ analysis of the mononuclear cell infiltrate of primary malignant melanoma in the skin. *Clin Exp Immunol* **51**: 77–82

Ravaud A, Legrand E, Delaunay MM, Bussieres E, Coulon V, Cany L, Huet S, Verdier D, Kind M, Chomy F, Bui BN and Gualde N (1995) A phase I trial of repeated tumor-infiltrating lymphocyte (TIL) infusion in metastatic melanoma. *Br J Cancer* **71**: 331–336

Rice GE, Munro JM, Corless C and Bevilacqua MP (1991) Vascular and nonvascular expression of INCAM-110. A target for mononuclear leukocyte adhesion in normal and inflamed human tissues. *Am J Pathol* **138**: 385–393

Rohde D, Schluter-Wigger W, Mielke V, Von Denriesch P, Von Gaudecker B and Sterry W (1992) Infiltration of both T cells and neutrophils in the skin is accompanied by the expression of endothelial leukocyte adhesion molecule-1 (ELAM-1): an immunohistochemical and ultrastructural study. *J Invest Dermatol* **98**: 794–799

Rosenberg SA, Spiess P and Lafreniere R (1986) A new approach to the adoptive immunotherapy of cancer with tumor infiltrating lymphocytes. *Science* **233**: 1318–1321

Rosenberg SA, Packard BS, Aebersold PM, Solomon D, Topalian SL, Toy ST, Simon P, Lotze MT, Young JC, Seipp CA, Simpson C, Carter C, Bock S, Swartzentruber D, Wei JP and White DE (1988) Special report: use of tumor infiltrating lymphocytes and interleukin-2 in the immunotherapy of patients with metastatic melanoma. A preliminary report. *N Engl J Med* **319**: 1676–1680

Salmi M, Granfors K, Leirisalo-Repo M, Hamalainen M, MacDermott R, Leino R, Havia T and Jalkanen S (1992) Selective endothelial binding of interleukin-2-dependent human T-cell lines derived from different tissues. *Proc Natl Acad Sci USA* **89**: 11436–11440

Salmi M, Grenman R, Grenman S, Nordman E and Jalkanen S (1995) Tumor endothelium selectively supports binding of IL-2-propagated tumor-infiltrating lymphocytes. *J Immunol* **154**: 6002–6012

Schieferdecker HL, Ullrich R, Weiss-Breckwoldt AN, Schwarting R, Stein H, Riecken E-O and Zeitz M (1990) The HML-1 antigen of intestinal lymphocytes is an activation antigen. *J Immunol* **144**: 2541–2549

Schmitt-Verhulst A (1994) Molecular basis of Immunity 1994: summary of sessions at the 1994 ENII Conference. *Res Immunol* **145**: 297–314

Schweighoffer T, Tanaka Y, Tidswell M, Erle DJ, Horgan KJ, Luce GE, Lazarovits AI, Buck D and Shaw S (1993) Selective expression of integrin a4b7 on a

© Cancer Research Campaign 1997

subset of human CD4+ memory T cells with hallmarks of gut-trophism. *J Immunol* **151**: 717–729

Shaw S, Luce GEG and Gilks WR (1994) Leucocyte differentiation antigen database. In *Leucocyte Typing, V*, Schlossman SF, Boumsell L, Gilks WR, Harlan JM, Kishimoto T, Morimoto, Ritz J, Shaw S, Silverstein RL, Springer TA, Tedder TF and Todd RF (eds), pp. 1172–1182. Oxford University Press: Oxford

Shimizu Y, Van Seventer GA, Horgan KJ and Shaw S (1990*a*) Costimulation of proliferative responses of resting CD4+ T cells by the interaction of VLA-4 and VLA-5 with fibronectin or VLA-6 with laminin. *J Immunol* **145**: 59–67

Shimizu Y, Van Seventer GA, Horgan KJ and Shaw S (1990*b*) Roles of adhesion molecules in T cell recognition: fundamental similarities between four integrins on resting human T cells (LFA-1, VLA-4, VLA-5, VLA-6) in expression, binding, and costimulation. *Immunol Rev* **114**: 109–143

Shimizu Y, Van Seventer GA, Horgan KJ and Shaw S (1990*c*) Regulated expression and function of three VLA (b1) integrin receptors on T cells. *Nature* **345**, 250–253

Shimizu Y, Newman W, Gopal TV, Horgan KJ, Graber N, Beall LD, Van Seventer GA and Shaw S (1991*a*) Four molecular pathways of T cell adhesion to endothelial cells: roles of LFA-1, VCAM-1 and ELAM-1 and changes in pathway hierarchy under different activation conditions. *J Cell Biol* **113**: 1203–1212

Shimizu Y, Shaw S, Graber N, Gopal TV, Horgan KJ, Van Seventer GA and Newman W (1991*b*) Activation-independent binding of human memory T cells to ELAM-1. *Nature* **349**: 799–802

Shimizu Y, Newman W, Tanaka Y and Shaw S (1992) Lymphocyte interactions with endothelial cells. *Immunol Today* **13**: 106–112

Spertini O, Luscinskas FW, Kansas GS, Munro JM, Griffin JD, Gimbrone MA Jr and Tedder TF (1991) Leukocyte adhesion molecule-1 (LAM-1, L-selectin) interacts with an inducible endothelial cell ligand to support leukocyte adhesion. *J Immunol* **147**: 2565–2573

Spiess P, Yang J and Rosenberg SA (1987) In vivo antitumor activity of tumor infiltrating lymphocytes expanded in recombinant interleukin-2. *J Natl Cancer Inst* **79**: 1067–1075

Springer TA (1994) Traffic signals for lymphocyte recirculation and leukocyte emigration: the multistep paradigm. *Cell* **76**: 301–314

Swerlick RA, Lee KH, Li L, Sepp NT, Caughman SW and Lawley TJ (1992) Regulation of vascular cell adhesion molecule 1 on human dermal microvascular endothelial cells. *J Immunol* **149**: 698–705

Takada Y, Elices MJ, Crouse C and Hemler ME (1989) The primary structure of the alpha-4 subunit of VLA-4 – homology to other integrins and a possible cell–cell adhesion function. *EMBO J* **8**: 1361–1368

Tanaka Y, Albelda SM, Horgan KJ, Van Seventer GA, Shimizu Y, Newman W, Hallam J, Newman PJ, Buck CA and Shaw S (1992) CD31 expressed on distinctive T cell subsets is a preferential amplifier of b1 integrin-mediated adhesion. *J Exp Med* **176**: 245–253

Tanaka Y, Adams DH, Hubscher S, Hirano H, Siebenlist U and Shaw S (1993) T-cell adhesion induced by proteoglycan-immobilized cytokine MIP-1b. *Nature* **361**: 79–82

Tugores A, Alonso MA, Sanchez-Madrid F and De Landazuri MO (1992) Human T cell activation through the activation-inducer molecule CD69 enhances the activity of transcription factor AP-1. *J Immunol* **148**: 2300–2307

Van Der Bruggen P, Traversari C, Chomez P, Lurquin C, De Plaen E, Van Den Eynde B, Knuth A and Boon T (1991) A gene encoding an antigen recognised by cytolytic T lymphocytes on a human melanoma. *Science* **2**: 1643–1647

Van Seventer GA, Shimizu Y, Horgan KJ and Shaw S (1990) The LFA-1 ligand ICAM-1 provides an important costimulatory signal for T cell receptor-mediated activation of resting T cells. *J Immunol* **144**: 4579–4586

Whiteside TL and Herberman RB (1992) Extravasation of antitumor effector cells. *Invasion Metastàsis* **12**: 128–146

Xu DS, Sorrell MF, Clemens DL, Casey CA and Tuma DJ (1994) Effects of ethanol feeding on the interaction of rat hepatocytes with laminin peptides. *Alcoholism – Clinical and Experimental Research* **18**: 1215–1219

Yannelli JR (1991) The preparation of effector cells for use in the adoptive cellular therapy of cancer. *J Immunol Methods* **139**: 1–16

Yoong KF and Adams DH (1996) Tumour infiltrating lymphocytes: insights into tumour immunology and potential for immunotherapy. *J Clin Molec Pathol* (in press)

Yoong KF, Williams A, Hubscher SG, Salmi M, Jalkanen S and Adams DH (1996) Expression of endothelial adhesion molecules and T-cell homing receptors in human hepatic-tumors. *FASEB J* **10**: 2473

© Cancer Research Campaign 1997